# Preoperative immune checkpoint inhibition and cryoablation in early-stage breast cancer

**DOI:** 10.1016/j.isci.2024.108880

**Published:** 2024-01-12

**Authors:** Elizabeth Comen, Sadna Budhu, Yuval Elhanati, David Page, Teresa Rasalan-Ho, Erika Ritter, Phillip Wong, George Plitas, Sujata Patil, Edi Brogi, Maxine Jochelson, Yolanda Bryce, Stephen B. Solomon, Larry Norton, Taha Merghoub, Heather L. McArthur

**Affiliations:** 1 Breast Medicine Service, Department of Medicine, Memorial Sloan Kettering Cancer Center, New York, NY, USA; 2 Ludwig Collaborative and Swim Across America Laboratory, Department of Pharmacology and Mayer Cancer Center, Weill Cornell Medicine, New York, NY, USA; 3 Computational Oncology Service, Department of Epidemiology & Biostatistics, Memorial Sloan Kettering Cancer Center, New York, NY, USA; 4 Earle A. Chiles Research Institute, Robert W. Franz Cancer Center, Providence Cancer Institute, Portland, OR, USA; 5 Immune Monitoring Core Facility, Ludwig Center for Cancer Immunotherapy, Memorial Sloan Kettering Cancer Center, New York, NY, USA; 6 Breast Surgery, Department of Medicine, Memorial Sloan Kettering Cancer Center, New York, NY, USA; 7 Department of Epidemiology and Biostatistics, Memorial Sloan Kettering Cancer Center, New York, NY, USA; 8 Department of Pathology, Memorial Sloan Kettering Cancer Center, New York, NY, USA; 9 Department of Radiology, Memorial Sloan Kettering Cancer Center, New York, NY, USA; 10 Department of Medicine, Division of Medical Oncology, University of Texas Southwestern Medical Center, Dallas, TX, USA

**Keywords:** Health sciences, Oncology, Immunology

## Abstract

Local cryoablation can engender systemic immune activation/anticancer responses in tumors otherwise resistant to immune checkpoint blockade (ICB). We evaluated the safety/tolerability of preoperative cryoablation plus ipilimumab and nivolumab in 5 early-stage/resectable breast cancers. The primary endpoint was met when all 5 patients underwent standard-of-care primary breast surgery undelayedly. Three patients developed transient hyperthyroidism; one developed grade 4 liver toxicity (resolved with supportive management). We compared this strategy with cryoablation and/or ipilimumab. Dual ICB plus cryoablation induced higher expression of T cell activation markers and serum Th1 cytokines and reduced immunosuppressive serum CD4^+^PD-1^hi^ T cells, improving effector-to-suppressor T cell ratio. After dual ICB and before cryoablation, T cell receptor sequencing of 4 patients showed increased T cell clonality. In this small subset of patients, we provide preliminary evidence that preoperative cryoablation plus ipilimumab and nivolumab is feasible, inducing systemic adaptive immune activation potentially more robust than cryoablation with/without ipilimumab.

## Highlights

• Preoperative cryoablation plus ipilimumab and nivolumab is feasible in breast cancer
• This therapy induces systemic adaptive immune activation in patients
• This therapy is potentially more robust than cryoablation with/without ipilimumab

## Graphical abstract

**Figure:**
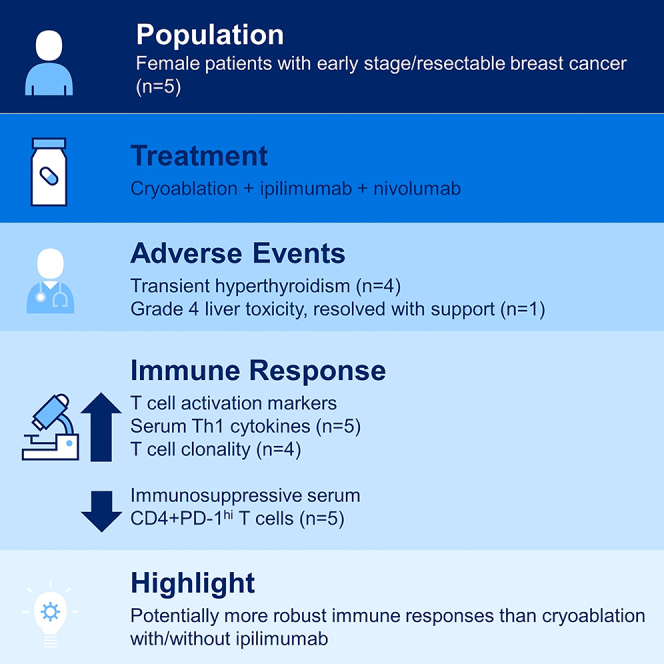

## Introduction

Over the last 10 years, the development of immune checkpoint blockade (ICB) drugs that target cytotoxic T lymphocyte-associated antigen (CTLA-4), programmed cell death receptor 1 (PD-1), or programmed cell death ligand 1 (PD-L1) have significantly improved outcomes across a number of cancers including melanoma, bladder cancer, lung cancer, and Hodgkin’s lymphoma.^1^^,^^2^ Initial studies indicated that cancers with higher burden of non-synonymous mutations that engender neoantigen presentation are more likely to respond to ICB.^3^ Historically, breast cancer was perceived to be less inherently immune-responsive, due in part to modest mutational burden. Consequently, early efforts with ICB largely focused on the triple-negative and human epidermal growth factor receptor 2-positive (HER2+) subtypes, which can be associated with higher lymphocyte infiltration and higher mutational burden compared with the hormone receptor-positive (HR+) subtype.^4^

Correlative science from early ICB monotherapy studies suggested that “cold” breast cancers devoid of CD8^+^ T cells may be less inherently responsive to ICB.^5^ However, response rates may be improved when ICB is combined with specific systemic therapies or local tumor-ablative strategies. For example, palliative radiation combined with pembrolizumab-mediated PD-1-directed ICB conferred durable responses outside the radiation field among women with pre-treated metastatic triple-negative breast cancer in a modestly powered study.^6^ Moreover, pembrolizumab was recently approved by the United States FDA in combination with chemotherapy (nab-paclitaxel, paclitaxel, or gemcitabine/carboplatin) for advanced PD-L1-positive triple negative breast cancer (TNBC), after a progression-free survival advantage was observed in a large randomized phase 3 study.^7^ Additionally, the benefit from ICB is increased when administered earlier in the course of the disease, with improved pathologic complete responses observed in the neoadjuvant setting with ICB and chemotherapy combinations, regardless of PD-L1 status.^8^^,^^9^ Indeed, pembrolizumab is also now used the neoadjuvant setting in some patients with triple negative disease.^8^

Strategies that incorporate treatments such as radiotherapy and cryoablation act as an *in situ* vaccination, releasing tumor antigens and improving antigen presentation to convert immunogenically “cold” tumors to “hot.”^10^^,^^11^ Thus, these therapies may optimize responses to ICB. We have specifically explored how cryoablation enhances immune response. We previously investigated the immunogenic impact of single-agent ICB using ipilimumab (10 mg/kg) and/or cryoablation in a pilot study in 18 women with early-stage breast cancer (of any subtype).^10^ The study demonstrated that ipilimumab, cryoablation, and the combination were safe and well tolerated, and all patients underwent standard of care definitive surgery without complications or delays. Furthermore, no treatment-associated grade 3 or 4 adverse events (AEs) were noted. T cell receptor (TCR) sequencing of tumor and blood samples at multiple time points indicated that the combination of ipilimumab and cryoablation diversified the TCR repertoire and increased the number of T cell clones both within the peripheral blood and tumor, thereby indicating T cell expansion in response to increased antigen presentation.^12^ Patients who received ipilimumab plus cryoablation had sustained activation and proliferation of CD4^+^ and CD8^+^ T cells in the periphery.

Recent data suggest that nivolumab, an anti-PD-1 antibody, synergizes with ipilimumab in other cancers.^13^ Moreover, a recent study from our group identified a suppressive CD4^+^ T cell subset that expresses high levels of PD-1 (CD4^+^ Foxp3^–^ T cells expressing PD-1, termed 4PD-1^hi^). 4PD-1^hi^ cells dampen T cell response and infiltrate tumors in proportion to tumor burden. We showed that combined blockade with ipilimumab and nivolumab led to a decrease in 4PD-1^hi^ cells, improving antitumor activity.^14^

Here we sought to evaluate the safety and tolerability of preoperative, single-dose ipilimumab and nivolumab in combination with cryoablation in patients with early-stage/resectable breast cancer. Safety was defined as the absence of an AE necessitating a delay in primary breast surgery. Secondary aims of the study were to explore and characterize pre- and post-intervention peripheral and tumor responses to ipilimumab, nivolumab, and cryoablation. To understand the immunogenic impact of dual versus single agent ICB with cryoablation, we also compared peripheral immune activation with results from the prior pilot study.^10^

## Results

### Study population

Five patients were enrolled in the study. Baseline characteristics are outlined in Table 1. The median age at diagnosis was 54 (range 51–65) years. All 5 patients had ER+, HER2– breast cancer. Two patients (patients 3 and 5) received adjuvant chemotherapy (dose-dense doxorubicin with cyclophosphamide followed by paclitaxel) for node-positive disease. After a median follow-up of 47 months, all patients are alive without any evidence of metastatic breast cancer since their original breast cancer diagnosis. Patient 1 was lost to follow-up after 15 months and received endocrine care outside of MSK. Patient 2 declined adjuvant radiation and endocrine therapy; she developed a local recurrence 47 months after her original breast cancer diagnosis. Patient 3 remains actively followed for over 49 months since diagnosis. At 33 months, she developed a primary stage 1 lung cancer treated with wedge resection. She has no evidence of recurrence of either breast or lung cancer. Patient 4 developed a high-grade myxofibrosarcoma in her lower extremity 29 months after her original breast cancer diagnosis. Treatment included surgical excision and radiation. Patient 4 was subsequently found to have a *BRCA2* mutation and remains free of disease to date at 48 months after diagnosis. Patient 5 remains free of disease 43 months after diagnosis.

**Table 1:** Characteristics of patients treated with ipilimumab, nivolumab, and cryoablation (n = 5)

Patient ID	1	2	3	4	5
Age (years)	54	54	53	63	51
Pathologic stage	pT1aN0	pT1cN0(i^+^)	pT2N1a	pT1cN0	pT1bN1
Subtype	ER+/HER2–	ER+/HER2–	ER+/HER2–	ER+/HER2–	ER+/HER2–
Time to surgery after ipilimumab and nivolumab-mediated ICB	10 days	10 days	12 days	11 days	9 days
Surgery	Mastectomy	Mastectomy	Mastectomy	Mastectomy	Lumpectomy
Adjuvant chemotherapy			X		X
Follow-up (months)	15	47	49	48	43

ER+, estrogen receptor-positive; HER2–, human epidermal grown factor receptor 2-negative.

### Safety and tolerability

The primary endpoint of this study was reached, as all 5 women underwent standard-of-care primary breast surgery without delay. However, patients 1, 4, and 5 developed grade 1 transient hyperthyroidism. Patient 1 was lost to follow-up, but at the last toxicity visit, her free T4 was normal. In patient 4, hyperthyroidism resolved without further intervention. Patient 5 developed hyperthyroidism 3 weeks after surgery, leading to a delay in an unplanned axillary dissection at the surgeon’s discretion. The patient ultimately decided not to proceed with the axillary dissection and her transient hyperthyroidism resolved without intervention after 6 weeks. Additionally, 8 weeks after primary breast surgery and 10 weeks after immunotherapy, patient 4 developed grade 4 liver toxicity. Prior to her diagnosis with breast cancer, she had a distant history of hepatic steatosis. At the time that she developed liver toxicity, she had been on exemestane for 4 weeks. She was admitted to the hospital and prescribed steroids and mycophenolic acid for 3 months. Liver biopsy showed no signs of immune-mediated hepatitis; this was complicated by the fact that she had received 24 h of steroids prior to biopsy. Pathologic review indicated that the injury pattern could be caused by drug-induced liver injury, including potentially from the exemestane, which was then discontinued. Five months later, once her liver function tests revealed amelioration of toxicity to grade 1, the patient was switched to tamoxifen. Initially, liver function tests performed over the first 3 months of treatment vacillated between normal and grade 1 elevations in aspartate aminotransferase (AST), alanine aminotransferase (ALT), and (alkaline phosphatase)ALP. Her most recent liver function tests were within the normal range. Repeat imaging of her liver is consistent with mild hepatic steatosis. AEs for all 5 patients are documented in Table 2.

**Table 2:** Adverse events among treated patients (n = 5)

Event	Grade 1	Grade 2	Grade 3	Grade 4
Cough	1			
Urticaria			1	
ALT increased	4	1	2	1
AST increased		1	1	
ALK increased	3			
Pruritis	3			
Bilirubin increased		1	2	1
Breast/chest wall pain	2			
Dry eye	1			
Hyperthyroidism	3			
Paresthesia	1			
Decreased platelet count	1			
Decreased WBC count	1			
Fatigue	1			
Rash	4			
Dyspnea	1			

ALK, alkaline phosphatase; ALT, alanine aminotransferase; AST, aspartate aminotransferase; WBC, white blood cell. Blank cells indicate no events.

### Characterization of T cells in peripheral blood

**Figure 1:**
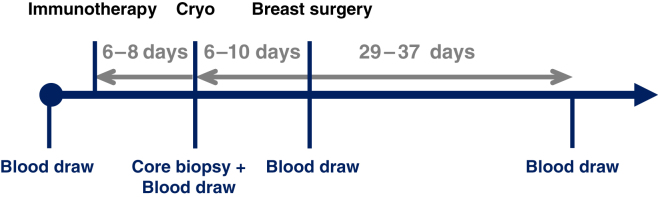
Study schema Combined immune checkpoint blockade (immunotherapy) was administered 1–5 days prior to, and cryoablation (Cryo) was performed 7–10 days prior to standard-of-care surgery. Toxicity evaluation continued for 12 weeks after drug administration. Blood for immune correlates was obtained at baseline, cryoablation, surgery, and 2–4 weeks thereafter (see Table S1 for individual patient timelines). Tumor samples were obtained at cryoablation and surgery.

**Figure 2:**
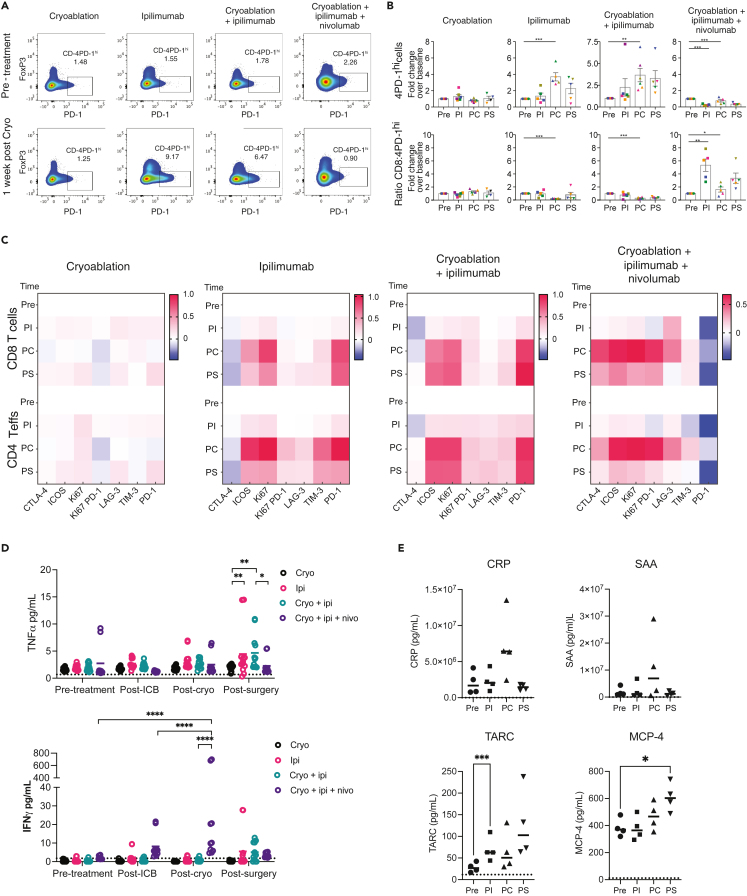
The combination of ipilimumab, nivolumab, and cryoablation induces T cell activation in the periphery (A) Representative bivariate plots of PD-1 vs. Foxp3 surface expression on CD3^+^CD4^+^ T cells at baseline and 2 weeks post-treatment from a single patient from each treatment arm to identify CD4^+^PD-1^hi^ T cells (4PD-1^hi^). (B) Quantitation of CD4^+^PD-1^hi^ T cells (top) and the ratio of CD8^+^ to 4PD-1^hi^ cells (bottom) in each treatment arm. Pre = baseline (pre-treatment), PI = post-immunotherapy, PC = post-cryoablation, PS = post-surgery. Cohort numbers are: cryoablation, n = 7; ipilimumab, n = 6; cryoablation plus ipilimumab, n = 6; cryoablation plus ipilimumab plus nivolumab, n = 5. (C) Heatmaps of expression of T cell activation markers in CD4^+^ T effector (Teff) cells and CD8^+^ T cells in each treatment arm. Data are represented as the average log fold-change (log fold change [FC]) relative to baseline (t = 0) for each time point. (D) Comparison of TNFα and IFNγ serum concentration between patients in each treatment arm. Statistics were calculated using two-way ANOVA (∗p < 0.05, ∗∗p < 0.01, ∗∗∗p < 0.001, ∗∗∗∗p < 0.0001). (E) Serum concentrations of CRP, SAA, TARC, and MCP-4 pooled from 4 patients at baseline and 1, 2, and 6 weeks post-treatment with ipilimumab, nivolumab, and cryoablation. Statistics were calculated using paired t tests: ∗p < 0.05, ∗∗p < 0.01, ∗∗∗p < 0.05. For all cytokine data, dotted lines represent lower limit of detection for each cytokine.

Localized treatments inducing tumor destruction, such as cryoablation have been shown to induce immunogenic cell death that leads to systemic activation of the adaptive immune system.^15^ We investigated whether combining ipilimumab and nivolumab with cryoablation led to any changes in the frequencies of T cells or their activation status in peripheral blood using multicolor FACS analysis of peripheral blood mononuclear cells (PBMCs) banked from each time point outlined in Figure 1 and Table S1. We compared immune changes in the current patient cohort to those in patients from our original trial who were treated with ipilimumab or cryoablation alone or the combination of both.^10^ There were no substantial changes in the frequencies of CD3^+^, CD8^+^, or CD4^+^Foxp3^–^ effector T cells in blood over baseline at any time after any treatment with ICB or cryoablation in this or the previous trial (Figure S1A). In general, cryoablation alone induced little to no changes in T cell frequencies or their activation in peripheral blood. In all patients treated with ipilimumab (alone or in combination with cryoablation), CD4^+^Foxp3^+^ regulatory T cells became more abundant at 2 weeks (PC), but these cells’ frequency started to decrease at 6 weeks (PS) post-baseline. Consistent with previous findings,^14^ we found that 4PD-1^hi^ immunosuppressive cells expanded in patients treated with ipilimumab alone or in combination with cryoablation (Figures 2A and 2B). However, in the current cohort of patients receiving ipilimumab and nivolumab plus cryoablation, there was a decrease in the 4PD-1^hi^ T cell population at weeks 1 (PI) and 6 (PS), which led to a corresponding increase in the effector-to-suppressor cell ratios (Figures 2A and 2B). As the anti-PD-1 antibody used for FACS does not compete with nivolumab, any changes in PD-1 expression on T cells (e.g., CD4^+^PD-1^hi^) cannot be attributed to an increase or decrease in the binding of the therapeutic antibody.^14^

Within the time points examined, T cell activation peaked at 2 weeks post-baseline (PC) in all cohorts treated with ICB. In all cohorts, ICOS and Ki67 expression increased in both CD4^+^ and CD8^+^ T cells (Figure 2C). However, in the cohort receiving ipilimumab, nivolumab, and cryoablation Ki67^+^PD-1^+^ CD4^+^ and CD8^+^ T cell populations increased. Ki67^+^PD-1^+^ CD8^+^ T cells have been previously described as re-invigorated T cells in response to anti-PD-1 treatment.^16^ In summary, the triple combination of ipilimumab and nivolumab plus cryoablation appears to promote a robust CD4^+^ and CD8^+^ T cell activation profile peaking around 2 weeks post-treatment.

Few viable cells were recovered from cryopreserved single-cell suspensions of tumor collected at surgery, preventing reliable analysis of immune infiltrates and their activation status in the tumors by FACS (Figure S1B).

We currently do not have data from breast cancer patients treated with ipilimumab plus nivolumab alone for comparison and to assess the contribution of cryoablation in this triple combination. However, we have obtained and analyzed flow cytometry data on banked PBMCs from previously published work on patients with urothelial cancer (UC) and melanoma treated with ipilimumab plus nivolumab on similar timelines.^17^ In addition, these patients experienced mixed clinical responses within the subsets of UC (n = 10; 2 complete response [CR], 3 partial response [PR], 1 stable disease [SD], 3 progression of disease [PD]) and melanoma (n = 10[; 6 PR, 3 SD, 1 PD]).

In the UC and melanoma cohorts receiving ipilimumab plus nivolumab UC and melanoma, there was an initial increase in 4PD-1^hi^ cells which decreased in subsequent time points to below baseline, while the cryoablation plus ipilimumab plus nivolumab cohort showed a decrease in 4PD-1^hi^ cells at all time points (Figure S2A). This resulted in an increase in CD4^+^ and CD8^+^ to 4PD-1^hi^ ratios in the cryoablation plus ipilimumab plus nivolumab cohort at all time points and in the UC and melanoma cohorts at later time points (e.g., 6 weeks post-treatment [PS]). There was also an increase in the frequency of Tregs in the blood of ipilimumab + nivolumab cohorts which persisted over time; this was less pronounced in the patients with breast cancer receiving cryoablation plus ipilimumab plus nivolumab. Additionally, there was an overall increase in CD4^+^ and CD8^+^ T cell activation markers (e.g., ICOS, Ki67 and, to a lesser extent, CTLA-4, Lag-3 and, Tim-3) in the ipilimumab plus nivolumab cohorts, which peaked 1 week post treatment (PI) and decreased over time. The cryoablation plus ipilimumab plus nivolumab cohort of patients peaked at 2 weeks post treatment (PC) and then decreased over time. This suggests that the cryoablation therapy may influence T cell activation in this cohort (Figure S2B). There was a decrease in CD4^+^ and CD8^+^ T cell PD-1 expression in all cohorts over time. In summary, while there were some similarities in across all three cohorts, the addition of cryoablation plus ipilimumab plus nivolumab appeared to influence the magnitude and kinetics of T cell activation. However, we cannot exclude that these differences might be cancer specific (e.g., breast cancer vs. UC/melanoma).

### Peripheral Th1 and Th2 cytokine response

We compared serum cytokine levels in the current study to those in patients treated with either ipilimumab or cryoablation alone, or the combination of both. In general, ipilimumab alone increased serum levels of certain Th2 cytokines such as IL-10 and IL-13, whereas the combination of ipilimumab and nivolumab plus cryoablation increased Th1 cytokines (Figure S3). The most substantial changes between these patient cohorts were seen in TNFα and IFNγ. Serum TNFα increased in patients treated with either ipilimumab alone or cryoablation plus ipilimumab starting at 1 week (PI) and peaked at 6 weeks (PS) (Figure 2D), but this increase was not observed in patients treated with the combination of cryoablation plus ipilimumab and nivolumab. The combination of cryoablation plus ipilimumab and nivolumab substantially increased IFNγ in the serum 6 weeks (PS). Thus, it appears that each of these treatment regimens may have differential effects on serum cytokine levels with distinct kinetics.

In addition to the Th1/Th2 cytokines analyzed previously, we also evaluated plasma from patients in the current study for additional cytokines and pro-inflammatory factors over time. In patients receiving ipilimumab and nivolumab plus cryoablation, levels of several pro-inflammatory factors such as c-reactive protein (CRP) and serum amyloid A (SAA) increased after cryoablation (2 weeks [PC]) (Figure 2E). Pro-inflammatory cytokines such as thymus and activation-regulated chemokine (TARC, CCL17) and monocyte chemoattractant protein-4 (MCP-4) also increased at weeks 1 (PI) and 6 (PS), respectively.

### Characterization of the T cell repertoire in the tumor and periphery

**Figure 3:**
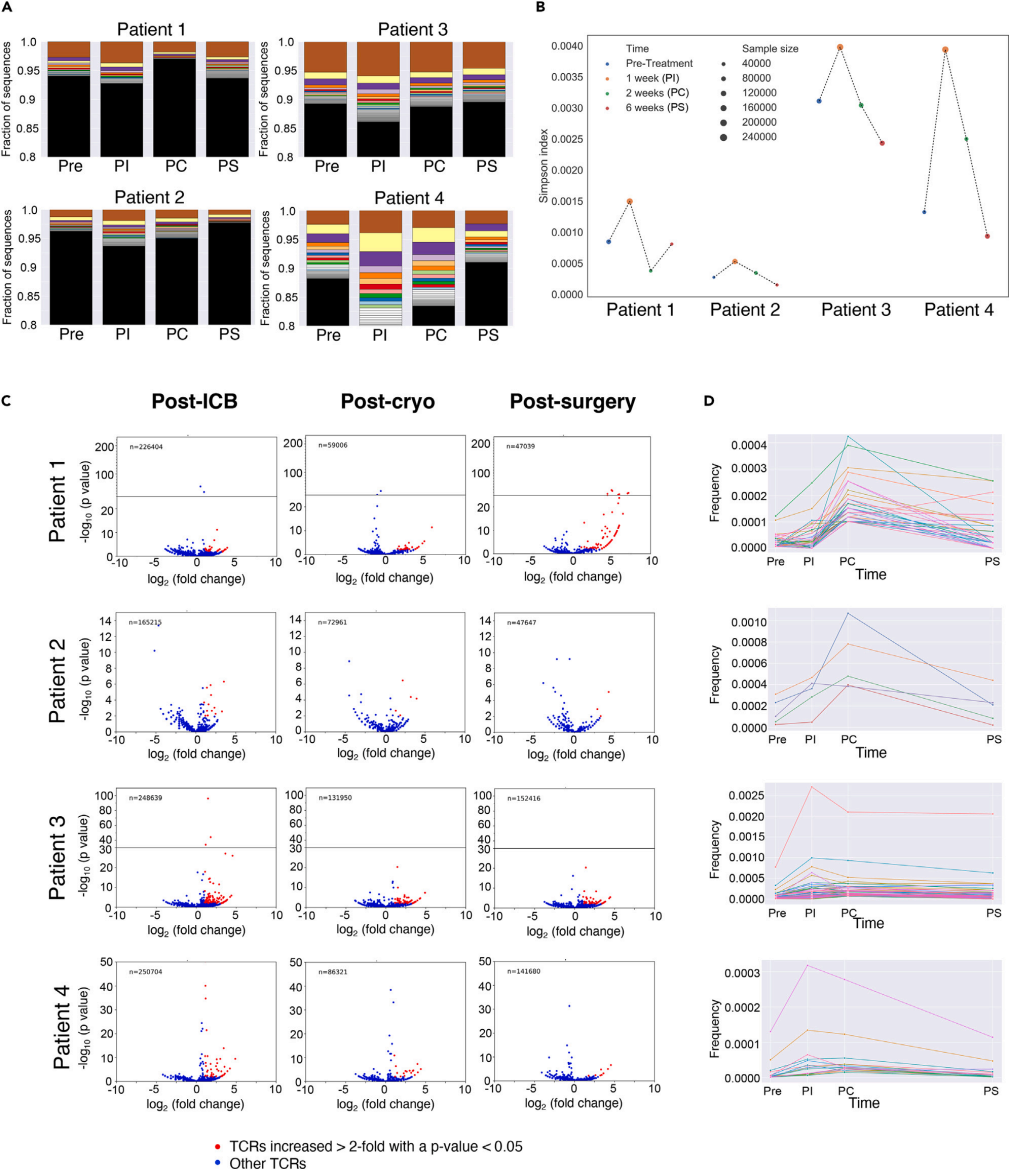
T cell receptor (TCR) sequencing analysis of blood samples pre- and post-treatment (A) Frequencies of T cell clones ranked and color-coded, with the most abundant clones in color. (B) Simpson index for each time point. (C) Volcano plots of log_2_ fold-change (Fc) versus -log_10_ p value vs. pre-treatment. Lines through the y axis indicate a change in p value scale. (D) Frequency over time of the clones in the blood that expanded at 2 weeks compared with pre-treatment.

We performed TCR sequencing in the 4 patients for whom samples were available to determine whether the combination of ipilimumab and nivolumab plus cryoablation alters the T cell repertoire in tumor or blood. In all 4 patients, the repertoire became more clonal following treatment with ipilimumab and nivolumab (Figure 3A, week 1 (PI) vs. 0 (Pre), as indicated by the relative size of the largest clones. This effect appeared to be transient, decreasing at later time points.

The dominance and kinetics of the top clones in the blood was mirrored by TCR clonality at these time points as measured by the Simpson index (Figure 3B). In all 4 patients, TCR clonality increased at week 1 (PI) and reverted to baseline at later time points. Thus, these changes in clonality can be attributed to treatment with ipilimumab and nivolumab but not cryoablation. On an individual basis, patients 1 and 2 had more diverse (less clonal) TCR repertoires while patients 3 and 4 had less diverse repertoires (more clonal). Patient 3 had a very dominating large clone at all time points. Patient 4 had an abnormally high clonal population of T cells that dominated the top 20% of T cells in the blood and had the largest increase in clonality following the treatment.

We found substantial expansion of at least some T cell clones in blood at every time point in all patients (Figure 3C, red dots). Patient 1 had the most expanded T cell clones at 6 weeks, while patients 3 and 4 had the most expanded clones at 1 week (PI), indicating earlier response. Patient 2 had the fewest expanded clones, which may suggest less immune response. Tracking the frequency of clones that substantially expanded at 2 weeks (PC), likely reflecting response to cryoablation, over time revealed that these clones peaked at 2 weeks (PC) in patients 1 and 2, while they peaked at 1 week (PI) in patients 3 and 4 (Figure 3D).

Overall, the blood repertoire tells a different story for each patient, underscoring inter-person heterogeneity. Patient 1 had low clonality pre-treatment and robust clone expansion following the cryoablation. Patient 2 had similar clonality before treatment, but a less specific response. Patient 5 had one dominating clone and little change in other clones following treatment. Patient 4 also started with low clonality due to a few large clones, but displayed large expansion of these clones following treatment, reverting to the original state by week 6 (PS).

We also sequenced samples from the tumor (5 locations, collected PC) and surrounding tissues for TCR repertoire. In general, fewer productive TCRs were detected in tumor and surrounding tissue samples compared with blood and their frequency varied substantially among sites, making it difficult to draw conclusions (Table S2). We did not observe any shared trends in clonal frequencies, clonality, or expansion of clones among patients. Patient 1 had a dominant clone in most tissue samples, most abundant in the pre-treatment biopsy (Figure S4A), that did not appear in blood, indicating a tissue-resident clone. This patient also had the highest clonality score in the pre-treatment sample (Figure S4B). A few TCR clones in the core and surrounding tissues expanded PC (Figure S4C).

Morisita overlap index analysis, which is biased toward large clones,^18^ revealed little overlap between clones present in tumor and normal breast tissue and blood (Figure S5). The overlap also varied on a patient-by-patient basis. Patients 1 and 3 showed an increase in overlap between blood and tumor after cryoablation, while patients 2 and 4 showed the opposite. Patient 1 showed the most overlap between Pre and PC core and tissue samples (Figure S5, green arrow).

## Discussion

In this pilot study, consistent with our primary aim, we demonstrated that the combination of ipilimumab, nivolumab, and cryoablation is feasible and can be integrated into preoperative treatment strategies without an AE delaying primary breast surgery. Cryoablation is believed to kill tumor cells via immunogenic cell death, releasing tumor-associated antigens that prime an immune response to the cancer, thereby serving as an *in situ* vaccine. While the implementation of immunotherapy has accelerated broadly across other cancer types, strategies that enhance immune responses and overcoming immune escape and acquired resistance remain elusive in breast cancer. For example, atezolizumab in combination with abraxane was originally granted accelerated approval for PD-L1-positive metastatic triple-negative breast cancer.^6^ More recently, however, atezolizumab was withdrawn from the market after a similar phase III study evaluating atezolizumab plus paclitaxel failed to meet the primary endpoint of progression-free superiority.^19^ At the same time, the demonstration that the addition of neoadjuvant pembrolizumab to chemotherapy for high-risk triple-negative breast cancer patients improves pathologic complete response rates and event-free survival^7^ generates hope that amplifying the adaptive immune response to breast cancer is possible.

The varying effects of combining immunotherapy and chemotherapy underscore the need for novel techniques to enhance immunotherapies. We hypothesized that the addition of ipilimumab and nivolumab further enhances the immune response to the cancer locally and systemically. Neither this study of dual ICB nor our original study of single-agent ipilimumab and cryoablation were designed to assess clinical benefit; however, after 74 months median follow-up, there has been no evidence of metastatic disease among all 18 patients treated on the original study, including the 3 patients with TNBC. In 2015, at the time of the original trial design for the present study, the goal was to include women with HR+ disease based on the original aforementioned study effort evaluating cryoablation and ipilimumab across multiple subtypes (including HR+ disease). These original patients, including patients with HR+ disease, continued to have largely favorable long-term event-free survival, and no concerning safety signals occurred. However, as the toxicity profiles were further elucidated, the nivolumab dose was modified to a flat dose in subsequent research trials, and patients with different cancer subtypes were included. Specifically, this work informed the ongoing phase 2 trial evaluating clinical benefit of ipilimumab, nivolumab, and cryoablation and survival outcomes exclusively in women with triple-negative breast cancer at high risk of recurrence.

With respect to strategies such as radiation versus cryoablation to enhance antigen presentation, the combination of immunotherapy with cryoablation (as opposed to radiation) in this study was chosen based on our earlier work and pre-clinical data available at the time. Specifically, pre-clinical and the aforementioned original clinical data indicated that cryoablation freezes macromolecules in their intact form and thereby preserves their antigenicity when thawed. Ionizing radiation causes degradation of these molecules so—at least theoretically—may be less antigenic.^10^ Furthermore, the combination of ipilimumab and cryoablation recapitulated our original study demonstrating systematic clonal expansion in response to cryoablation induced antigen presentation.^12^ To date, there is no head-to-head data comparison of cryoablation versus radiation in enhancing antigen presentation.

Secondary aims of the study were to explore and characterize pre- and post-intervention peripheral blood and tumor responses to the combination of ipilimumab, nivolumab, and cryoablation. We compared our immune monitoring data to those of the previous study^10^ and demonstrate stronger activation of T cells in the blood of patients treated with ipilimumab, nivolumab, and cryoablation compared with ipilimumab with or without cryoablation. In addition, we showed that the immune responses and kinetics in patients with breast cancer treated with cryoablation plus ipilimumab plus nivolumab were different from those of patients with UC or melanoma treated with ipilimumab plus nivolumab. This suggests that T cell activation is most pronounced when both ipilimumab and nivolumab are combined with cryoablation. The major caveat to this conclusion is that sample size is limited, and we did not evaluate nivolumab alone, nivolumab and ipilimumab, or cryoablation and nivolumab, which limits our understanding of the respective contribution of each agent.

In this study, we found dynamic changes in the population of 4PD-1^hi^ immunosuppressive T cells in the blood. The abundance of these cells decreased in patients treated with ipilimumab and nivolumab plus cryoablation, which increased the effector-to-suppressor cell ratio. In contrast, the size of this population notably increased in patients treated with treated with ipilimumab alone or in combination with cryoablation. This difference in effects on 4PD-1^hi^ activity between single-agent anti-CTLA-4 and the combination of anti-CTLA-4 with anti-PD-1 mirrors previously published data.^2^ Whereas CTLA-4 blockade stimulates the accumulation of 4PD-1^hi^ cells in the tumor and periphery, PD-1 blockade opposes 4PD-1^hi^ activity even in combination with anti-CTLA-4, thereby promoting a stronger antitumor immune response.^2^ While we found that the combination of ipilimumab and nivolumab plus cryoablation decreased the 4PD-1^hi^ T cell population at weeks 1 (PI) and 6 (PS), further studies are necessary to better characterize these effects over time, as well as their functional significance. The current findings suggest that the combination of ipilimumab and nivolumab engenders a uniquely potent immune response to cryoablation, given both the activation of effector T cells in the periphery and the mitigation of immunosuppressive 4PD-1^hi^ cells.

Patients receiving ipilimumab, nivolumab, and cryoablation showed increased levels of several pro-inflammatory factors in the blood at weeks 2 (PC) and 6 (PS). The release of pro-inflammatory Th1 cytokines has been strongly linked to antitumor immunity and is known to be promoted by immunotherapies.^20^^,^^21^ These cytokines are predominantly produced by activated T cells and have been linked with potent immune responses to both pathogens and cancer. While these cytokines can influence the activation of other immune cell types such as macrophages to enhance their effector function (e.g., phagocytosis), they can also directly inhibit tumor growth. IFNγ has been strongly associated with anti-tumor efficacy in preclinical and clinical studies;^21^ however, the role of TNFα in promoting tumor immunity is more ambiguous and most likely context dependent. Two studies examining serum TNFα levels after ICB treatment have drawn opposite conclusions in terms of clinical responses. In a study of non-small cell lung cancer (NSCLC),^22^ patients treated with nivolumab or pembrolizumab showed an increase in serum levels of IFNγ, TNFα, as well as many other pro-inflammatory cytokines, which correlated in better responses to anti-PD-1 inhibition and longer survival. However, in a study of melanoma,^23^ there was a decrease in serum levels of TNFα in patients with CRs, PRs, and SD. In contrast, patients with PD showed an increase in levels of serum TNFα. In addition, there is preclinical evidence that combined ICB and TNFα blockade results in better anti-tumor responses and overall survival.^21^ Of note, in our study, we observed an increase in serum TNFα levels in patients treated with ipilimumab alone or cryoablation + ipilimumab at 6 weeks (PS) (Figure 2D) but this was not observed in the group receiving cryoablation alone or the group receiving cryoablation plus ipilimumab plus nivolumab. Furthermore, only the group receiving cryoablation plus ipilimumab plus nivolumab showed an increase in serum IFNγ levels at weeks 1 (PI) and 2 (PC). Thus, TNFα and IFNγ likely have different kinetics depending on treatment rendered. The cytokine data provided in this study is limited to 5–7 patients per treatment group; thus we cannot conclude whether the roles of these cytokines correlate positively or negatively with clinical responses.

TCR sequencing and diversity analysis in 4 patients revealed that combined ICB using ipilimumab and nivolumab led to an increase in T cell clonality in blood soon after treatment (pre-cryoablation) in all patients, followed by a decrease to pre-treatment values. Traditionally anti-CTLA-4 is known to diversify (render less clonal) the TCR repertoire, while anti-PD-1 is known to make the repertoire more clonal.^24^ This suggests that the combination of ipilimumab and nivolumab in this cohort behaves similarly to treatment with anti-PD-1. As with most effects of immunotherapies, the change in clonality was transient. The frequencies and expansion of TCR clones in the blood and tumor varied on a patient-by-patient basis, limiting further conclusions about these factors given that only 4 patients were analyzed in this cohort.

In conclusion, the original study of ipilimumab and cryoablation and the present study combining them with nivolumab fundamentally contribute to our evolving understanding of how immunotherapy can be used to improve outcomes for breast cancer patients. We have demonstrated enhanced immune responses with the addition of nivolumab to ipilimumab and cryoablation in the subset of patients studied. Essential to improving outcomes for patients will be the thoughtful integration of immunotherapy with a refined understanding of both short- and long-term AEs. Moreover, it will be critical to elucidate the relationship between the timing of immune changes and durable systemic responses in patients. As a result of this trial and as a next step toward evaluating immunotherapy and clinical benefit in breast cancer patients, our ongoing phase 2 study of perioperative ipilimumab, nivolumab, and cryoablation (NCT03546686) will evaluate event-free survival in a larger cohort of patients at especially high risk of recurrence.

### Limitations of the study

There are several limitations to the work presented here. One major weakness is a small patient sample size which limits our ability to make broader inferences. In particular, this study is limited by the lack of diversity of the tumor subtypes given that all the included patients are ER+. All 5 patients who received dual ICB had ER+, HER2– breast cancer and 2 received adjuvant chemotherapy for axillary lymph node-positive disease. The heterogeneity in treatment and homogeneity of tumor type limit interpretations regarding biological and clinical outcome. Moreover, while the study met its primary endpoint of no delays of primary breast surgery, hyperthyroidism in one patient delayed a subsequently planned secondary axillary surgery. Further, patient 4 required a prolonged course of steroids in the setting of grade 4 liver toxicity, though this was not conclusively immune-related hepatitis, as she had already received several doses of steroids. As our understanding of immune-related toxicities and treatment for them evolves, it will be critical to identify patient populations for whom the toxicity risk versus potential benefit tips toward improving outcomes. To this end, our phase II trial includes only TNBC patients who do not achieve CR, and therefore have a substantially greater risk of distant recurrence and death. In this population, the potential benefits of such therapy may more significantly outweigh the risks of immune-related toxicities. To date, existing immunotherapies are more effective in triple negative breast cancer. Furthermore, we did not evaluate nivolumab alone, nivolumab and ipilimumab, or cryoablation and nivolumab, which limits our understanding of the respective contribution of each agent.

## STAR★Methods

### Key resources table

**Table:** 

REAGENT or RESOURCE	SOURCE	IDENTIFIER
**Antibodies**		

CD8-Qdot 605 (clone 3B5)	ThermoFisher Scientific	Cat# Q10009; RRID: AB_2556437
CD4-Qdot 655 (clone S3.5)	ThermoFisher Scientific	Cat# Q10007; RRID: AB_11180600
PD-1-PE (clone MIH4)	BD Pharmigen	Cat# 557946; RRID: AB_647199
LAG-3-FITC (clone 17B4)	Enzo Life Sciences	Cat# ALX-804-806F; RRID: AB_10997322
ICOS-PE-Cy7 (clone ISA-3)	ThermoFisher Scientific	Cat# 25-9948-42; RRID: AB_1518754)
TIM-3-APC (clone 344823)	R&D Systems	Cat# FAB2365A; RRID: AB_1964725
CD3-BV570 (clone UCHT1)	Biolegend	Cat# 100237; RRID: AB_2562039
Ki-67-AlexaFluor700 (clone B56)	BD Biosciences	Cat# 561277; RRID: AB_1061157
Foxp3-eFluor450 (clone PCH101)	eBioscience	Cat# 48-4776-41; RRID: AB_1834365
CTLA-4-PerCP-eFluor710 (clone 14D3)	eBioscience	Cat# 46-1529-42; RRID: AB_2573718
mouse IgG1κ-PE	BD Pharmingen	Cat# 555749; RRID: AB_396091
mouse IgG1- FITC	Enzo Life Sciences	Cat# ADI-SAB-600FI-050; RRID: AB_10997247
mouse IgG1κ-PE-Cy7	ThermoFisher Scientific	Cat# 25-4714-80; RRID: AB_657914
rat IgG2aκ-APC	R&D Systems	Cat# IC006A; RRID: AB_357254
mouse IgG2aκ-PerCP-eFluor710	eBioscience	Cat# 46-4724-82; RRID: AB_1834451

**Biological samples**		

Breast tissue from patients with early stage breast cancer	N/A	N/A
Blood samples from patients with early stage breast cancer	N/A	N/A

**Chemicals, peptides, and recombinant proteins**		

FACS buffer (PBS [phosphate-buffered saline] containing bovine 1% serum albumin and 0.05 mM EDTA	N/A	N/A
LIVE/DEAD™ Fixable Aqua Dead Cell Stain	Invitrogen	Cat# L34957
Foxp3/Ki-67 Fixation/Permeabilization Concentrate and Diluent	eBioscience	Cat# 00-5521-00

**Critical commercial assays**		

Human Proinflammatory Panel 10-plex kit	Meso Scale Diagnostics	Cat#s K15049D-1, K15047D-1, K15198D-1
DNeasy extraction kit	Qiagen	N/A
Adaptive hsTCRBv4 assay	Adaptive Biotechnologies	N/A

**Deposited data**		

Clinical trial registry number	ClinicalTrials.gov	NCT02833233
TCR clone abundance tables	Adaptive Biotechnologies	supplemental information (tsv files)

**Software and algorithms**		

FACSDiva software	BD Biosciences	N/A
FlowJo software	FlowJo LLC	version 10
MSD Discovery Workbench software	Meso Scale Diagnostics	N/A
Python	Python Software Foundation	N/A

**Other**		

Ice Pearl and Ice Force probes	Boston Scientific	N/A
Common Terminology Criteria for Adverse Events	National Cancer Institute	v4.0
LSRFortessa flow cytometer	BD Biosciences	N/A

### Resource availability

#### Lead contact

Further information and requests for resources and reagents should be directed to and will be fulfilled by the lead contact, Heather L. McArthur (heather.mcarthur@utsouthwestern.edu).

#### Materials availability

This study did not generate new unique reagents.

#### Data and code availability

• Anonymized patient data, as well as flow cytometry and serum cytokine data reported in this paper will be shared by the lead contact upon request. The raw TCR sequencing data is not available since Adaptive Biotechnologies does not release raw data and instead provides processed TCR clone abundance tables. We have provided these tables as tsv files in the supplemental information.
• This paper does not report original code.
• Any additional information required to reanalyze the data reported in this paper is available from the lead contact upon request.

### Experimental model and study participant details

Between December 2016 and August 2017, women with biopsy-proven invasive breast cancer planning curative-intent mastectomy or lumpectomy at Memorial Sloan Kettering Cancer Center (MSK) were enrolled in this single-arm study (NCT02833233). Eligible women ≥ 18 years of age, with ≥ 1.5-cm, histologically confirmed, invasive carcinoma of the breast and no evidence of metastases were identified at the time of the initial surgical consultation and enrolled after providing informed consent. Male patients with early-stage BC were not included as BC primarily affects female patients, and inclusion of this population would result in a low sample size and render the study insufficiently powered to control for variables related to biological sex. Participants were enrolled regardless of race (breakdown: 60% white, 20% Asian, 20% unknown) or ethnicity (breakdown: 20% Hispanic or Latino/a of any race; 80% non-Hispanic/Latino/a). At the time of this clinical trial, our institution did not have a way to track socioeconomic indicators; we are building toward the capacity to track and report on those data with the rollout of an Epic-based electronic health record in February 2025. Any HR/HER2 and nodal status were permitted. HR positivity was defined as ≥ 1% expression of either estrogen receptor (ER) or progesterone receptor by immunohistochemistry (IHC). HER2 positivity was defined as either 3+ expression by IHC and/or ≥ 2.0 HER2 to chromosome 17 centromere signals by the FISH test. Multifocal, multicentric, and synchronous bilateral invasive disease was permitted. Patients were excluded from the study if they had a known autoimmune disease and/or were on immunosuppressants or steroids. Surgery and related management activities were scheduled per standard of care. Research study appointments and interventions did not interfere with planned standard-of-care surgery. All available imaging was reviewed to determine eligibility for magnetic resonance imaging (MRI)- or ultrasound (US)-guided cryoablation. Patients were advised to discontinue any non-steroidal anti-inflammatory medications from the day of trial entry until 30 days after surgery, unless recommended by study investigator or required for the treatment of immune-related AEs.

After providing informed consent, all 5 patients received ipilimumab and nivolumab 1–5 days prior to cryoablation, which was performed 7–10 days prior to primary breast surgery. At the time of cryoablation, at least three 9- or 12-gauge US-guided core biopsies of the primary breast tumor were obtained. If three core biopsies from each patient could not be obtained, patients were deemed ineligible. Two core breast specimens were provided to the MSK Department of Pathology for routine testing to confirm diagnosis and for immunohistochemical (IHC) staining. Additional core biopsy specimens were sent to the MSK Ludwig Center Immune Monitoring Core Facility for analyses reported herein. Patients then underwent safety assessments 2–3 weeks post-surgery (PS) and then every 2–3 weeks thereafter until at least 12 weeks post-immunotherapy (PI). Research blood samples were obtained at baseline, cryoablation, surgery, and at safety assessments after surgery (Figure 1). Tumor samples were obtained at cryoablation and surgery. This schedule mirrored our prior study of single agent ipilimumab with or without cryoablation.^10^ In the current study, ipilimumab was administered intravenously (IV) at a dose of 1 mg/kg infused over 90 minutes, combined with nivolumab at 3 mg/kg IV infused over 60 minutes. The 1 mg/kg ipilimumab dose was selected based on its better tolerability when combined with nivolumab.^25^

The study was performed in accordance with ethical principles of the Declaration of Helsinki and the International Conference on Harmonization of Good Practice and approved by the MSK Institutional Review Board.

### Method details

#### Safety and feasibility assessments

The primary objective of this pilot study was to establish the safety and feasibility of dual immune checkpoint blockade (ICB) and cryoablation prior to definitive primary breast surgery. As per our previously published study, the regimen was deemed safe if at least 5 of 6 subjects completed the intervention without incurring AEs that necessitated a delay in the pre-determined, standard-of-care surgery date. Toxicities were assessed using the National Cancer Institute (NCI) Common Terminology Criteria for Adverse Events v4.0 (CTCAE). Patients were followed clinically and with serum and blood assessments (complete blood count, comprehensive metabolic panel, thyroid-stimulating hormone) to confirm safety every 2–3 weeks for 12 weeks after immunotherapy administration to document any potential delayed immune-related AEs. When 5 women underwent surgery without incurring any AE-related delays, the primary endpoint was met and enrollment to the trial was stopped.

#### Cryoablation procedure

Percutaneous cryoablation was performed 7–10 days prior to planned primary breast surgery. Cryoablation was performed under MRI or US guidance with Ice Pearl and Ice Force probes (Boston Scientific, Natick, MA). The probes were placed in the tumor and 2 freeze-thaw cycles were performed, where each freeze step lasted approximately 10 minutes, then the probes were removed. Intermittent imaging was performed to ensure that the ice ball did not encroach upon the skin. Patients for whom 3 core biopsies could not be performed prior to cryoablation were deemed ineligible. A prophylactic dose of cefazolin was also administered to reduce risk of infection.

#### Peripheral blood and intratumoral lymphocyte isolation

Peripheral blood was obtained at the time of consent, time of biopsy/cryoablation, time of surgery, and at the safety follow-up visit 30 days PS. Research biopsies were obtained at the time of cryoablation and fresh tumor tissue was submitted immediately following surgery to the MSK’s Immune Monitoring Core Facility, where tumor-infiltrating lymphocytes (TILs) were extracted and cryopreserved.

#### Flow cytometry analysis

Flow cytometry staining (FACS) was performed on both peripheral blood mononuclear cells (PBMCs) and harvested TILs.^10^ Briefly, one million PBMCs and TILs were washed with 2 mL FACS buffer (PBS [phosphate-buffered saline] containing bovine 1% serum albumin and 0.05 mM EDTA), resuspended in 50 μL FACS buffer and stained with a fixable Aqua viability dye (Invitrogen) and a cocktail of antibodies to the following surface markers: CD8-Qdot 605 (Invitrogen, 3B5), CD4-Qdot 655 (Invitrogen, S3.5), PD-1-PE (BD, MIH4), LAG-3-FITC (Enzo Life Sciences, 17B4), ICOS-PE-Cy7 (eBioscience, ISA-3), TIM-3-APC (R&D Systems, 344823). Cells were next fixed and permeabilized with the Foxp3/Ki-67 Fixation/Permeabilization Concentrate and Diluent (eBioscience), and subsequently stained intracellularly with the following antibodies: CD3-BV570 (Biolegend, UCHT1), Ki-67-AlexaFluor700 (BD, B56), Foxp3-eFluor450 (eBioscience, PCH101), and CTLA-4-PerCP-eFluor710 (eBioscience, 14D3). Isotype controls included the appropriate fluorochrome-conjugated mouse or rat IgG1, IgG1 κ, or IgG2a κ antibodies (BD Pharmingen, eBioscience, Enzo Life Sciences, R&D Systems). Stained cells were detected using an LSRFortessa flow cytometer with FACSDiva software (BD Biosciences). Analyses were performed using FlowJo software (version 10, FlowJo LLC). The percentages of CD4^+^ Teff, CD4^+^ regulatory T cells (Treg) and CD8^+^ T cell subsets were calculated as a proportion of live CD3^+^ T cells for each time point. The percentage of 4PD-1^hi^ cells was determined as a proportion of live CD3^+^CD4^+^ cells. The percentage of T cells expressing activation markers (eg, Ki67+, PD1+ etc.) was determined using a matched isotype staining control for each cell type. To account for baseline variability across subjects, the effect of therapy was described as a log of the fold change of each marker relative to baseline. Ratios of effector T cells to regulatory T cells (Teff/Treg) and effector T cells to CD4+Foxp3-PD-1^hi^ were calculated by dividing the frequency of CD8^+^ T cells by the frequency of Foxp3+CD4^+^ T cells or CD4+Foxp3-PD-1^hi^, respectively. The MIH4 clone of anti-PD-1 antibody used for FACS analysis does not compete with and is not substantially cross-blocked by nivolumab.^2^

#### Serum cytokine analysis

Serum collected at baseline and weeks 1 (PI), 2 (post-cryoablation [PC]), and 6 (PS) following start of therapy was processed and banked. Cytokine measurements were performed with electrochemiluminescence immunoassays following manufacturer instructions using the V-PLEX validated Human Proinflammatory Panel 10-plex kit (for IL-2, IL-4, IL-6, IL-8, IL-10, IL-12, IFNγ, TNFα, IL-1β and IL-13), Human Chemokine Panel 10-plex kit (for eotaxin, eotaxin-3, IL-8, IP-10, MCP-1, MCP-4, MDC, MIP-1α, MIP-1β, TARC) and Human Vascular Injury Panel 4-plex kit (for SAA, CRP, VCAM-1, and ICAM-1) purchased from Meso Scale Diagnostics (MSD, Cat #K15049D-1, K15047D-1, K15198D-1). The raw data was measured as light intensity detected by instrument photodetectors upon application of electricity to the plate electrodes. Data was analyzed using the MSD Discovery Workbench software. A 4-parameter logistic fit calibration curve was generated for each analyte using the standards to calculate the concentration of each sample. Both serum cytokine levels and a log fold-change of each analyte relative to baseline were determined.

#### TCR sequencing

Blood PBMCs were collected at week 0 (Pre), during, and after cryoablation (study schema, Figure 1; Table S1). Tumor samples were collected as a biopsy pre-cryoablation and 1-week PC from the mastectomy or lumpectomy samples, which were separated into tumor core, 1 cm and 3 cm from the core, and normal tissue(s) when available. Genomic DNA was extracted from banked PBMCs at MSK using the DNeasy extraction kit (Qiagen), while DNA from formalin-fixed paraffin-embedded tissue curls was extracted by Adaptive Biotechnologies. PBMC samples were run at deep resolution and all tissue samples were run at survey resolution using the Adaptive hsTCRBv4 assay. This Adaptive hsTCRBv4 platform has greater sensitivity than the version used in the previous ipilimumab plus cryoablation trial.^12^

#### TCR analysis

TCR clones were defined from the Adaptive hsTCRBv4 assay based on their CDR3 amino acid sequence. A clone and count table were used for all further analysis. Repertoire diversity was assessed using the Simpson index, defined as the sum of the clone frequency squared divided by the frequency of all clones in a sample. This measure is equal to the probability of encountering the same clone twice while randomly sampling TCRs, and is higher for more clonal (less diverse) samples.

Overlap between any two samples was measured using the Morisita similarity index, defined as the sum of the product of the clone frequencies from the two samples, normalized by the sum of the Simpson index of both samples.^18^ Thus, larger clones contribute more to this measure because differences in small clones are less substantial. The Morisita index is 0 for two samples that do not share any clone and 1 for identical samples.

Clones were also analyzed for substantial expansion between multiple samples from the same patient. A clone was deemed as expanded if its frequency increased by more than 2-fold and the Fisher exact test p-value was <0.05. This approach identifies clones that have expanded beyond what can be expected from sampling noise and largely filters out large fluctuation in small clones.

### Quantification and statistical analysis

For flow cytometry and cytokine analyses where individual time points were compared, the p values were calculated using a paired t test. A p value of < 0.05 was considered statistically significant. For cytokine analysis where multiple treatment groups were compared, p values were calculated using 2-way ANOVA corrected for multiple comparisons. Results can be found in the Results section, as well as Figure 2A Representative bivariate plots of PD-1 vs. Foxp3 surface expression on CD3^+^CD4^+^ T cells at baseline and 2 weeks post-treatment from a single patient from each treatment arm to identify CD4+PD-1^hi^ T cells [4PD-1^hi^]; B. Quantitation of CD4+PD-1^hi^ T cells and the ratio of CD8^+^ to 4PD-1^hi^ cells in each treatment arm. Cohort numbers are: cryoablation, n=7; ipilimumab, n=6; cryoablation plus ipilimumab, n=6; cryoablation plus ipilimumab plus nivolumab, n= 5; C. Heatmaps of expression of T cell activation markers in CD4^+^ T effector [Teff] cells and CD8^+^ T cells in each treatment arm. Data is represented as the average log fold-change (log FC) relative to baseline (t=0) for each time point; D. Comparison of TNFα and IFNγ serum concentration between patients in each treatment arm. Statistics were calculated using 2-way ANOVA [∗p<0.05, ∗∗p<0.01, ∗∗∗p<0.001, ∗∗∗∗p<0.0001]. E. Serum concentrations of CRP, SAA, TARC, and MCP-4 pooled from 4 patients at baseline and 1, 2, and 6 weeks post-treatment with ipilimumab, nivolumab, and cryoablation. Statistics were calculated using paired t tests: ∗p<0.05, ∗∗p<0.01, ∗∗∗p<0.05), Figure S1A Heatmaps of T cell populations of the ratio of effector to suppressor T cells in each treatment group. Cohort numbers are: cryoablation, n=7; ipilimumab, n=6; cryoablation plus ipilimumab, n=6; cryoablation plus ipilimumab plus nivolumab, n=5; B) Banked single-cell suspensions of tumor-infiltrating lymphocytes isolated from the tumors were analyzed by flow cytometry. Shown are the frequencies of total CD3^+^, CD8^+^,–and CD4^+^ effector [CD4+Foxp3-] T cells, Tregs [CD4+Foxp3+], and 4PD1^hi^ [CD4+Foxp3-PD1^hi^] T cells at various tumor and tissue sites +/- standard error of samples pooled from 3-5 patients. Normal S/I, normal tissue pooled from superior and inferior samples. Statistics were calculated using Student’s t test: ∗p<0.05, ∗∗p<0.01, ∗∗∗p<0.05), Figure S2 (Flow cytometry data was obtained from published work^17^ on patients with UC [n=94] and melanoma [n=188] treated with ipilimumab plus nivolumab and a similar analysis was performed as in the cohort of patients with breast cancer cohort receiving cryoablation plus ipilimumab plus nivolumab: A) Heatmaps of T cell populations of the ratio of effector to suppressor T cells in each treatment group. Data is represented as the average log10 fold-change [log FC] relative to baseline [t=0, Pre] for each time point; B) Heatmaps of expression of T cell activation markers in CD4^+^ T effector [Teff] cells and CD8^+^ T cells in each cohort), and S3 (Heatmaps of serum Th1 and Th2 cytokines in serum in each treatment group. Data for each time point are represented as the average of log_10_ fold change [FC] relative to the baseline levels (pg/ml) for each cytokine. Cohort numbers are: cryoablation, n=7; ipilimumab, n=6; cryoablation plus ipilimumab, n= 6; cryoablation plus ipilimumab plus nivolumab, n=5).

To restate, the statistics for TCR analysis, repertoire diversity was assessed using the Simpson index, defined as the sum of the clone frequency squared divided by the frequency of all clones in a sample. Overlap between any two samples was measured using the Morisita similarity index, defined as the sum of the product of the clone frequencies from the two samples, normalized by the sum of the Simpson index of both samples.^18^ Clones were also analyzed for substantial expansion between multiple samples from the same patient. A clone was deemed as expanded if its frequency increased by more than 2-fold and the Fisher exact test p-value was <0.05. Results can be found in the Results section, as well as Figure 3A Frequencies of T cell clones ranked and color-coded; B. Simpson index for each time point; C. Volcano plots of log_2_ fold-change [Fc] versus -log_10_ p-value vs. pre-treatment; D. Frequency over time of the clones in the blood that expanded at 2 weeks compared with pre-treatment; n=4), Figure S4A Frequencies of T cell clones in the tumor ranked by abundance; B. Simpson index for each time point in the tumor; C. Volcano plots of log_2_ fold change (Fc) vs. negative log_10_ p-value of data compared with pre-treatment in blood), Figure S5 (Heatmaps of the calculated Morisita overlap index between blood and tissue; n=4), and Table S2 (Number of productive T cell receptors [TCRs] per patient sample; n=4).

### Additional resources

Clinical trial registry number: NCT02833233.

Link: https://clinicaltrials.gov/study/NCT02833233.

## Supplemental information

Document S1. Figures S1–S5 and Tables S1 and S2

Data S1. Metadata file for TCR clone abundance tables provided in the supplement
