# Supplementary material for: Preoperative immune checkpoint inhibition and cryoablation in early-stage breast cancer
Source: iScience. 2024 Jan 12;27(2):108880. doi: 10.1016/j.isci.2024.108880 (PMC10850740; doi:10.1016/j.isci.2024.108880)
Supplement: Document S1. Figures S1–S5 and Tables S1 and S2 [file mmc1.pdf]

iScience, Volume *27*

Supplemental information

Preoperative immune checkpoint inhibition

and cryoablation in early-stage breast cancer

Elizabeth Comen, Sadna Budhu, Yuval Elhanati, David Page, Teresa Rasalan-Ho, Erika Ritter, Phillip Wong, George Plitas, Sujata Patil, Edi Brogi, Maxine Jochelson, Yolanda Bryce, Stephen B. Solomon, Larry Norton, Taha Merghoub, and Heather L. McArthur

Figure S1

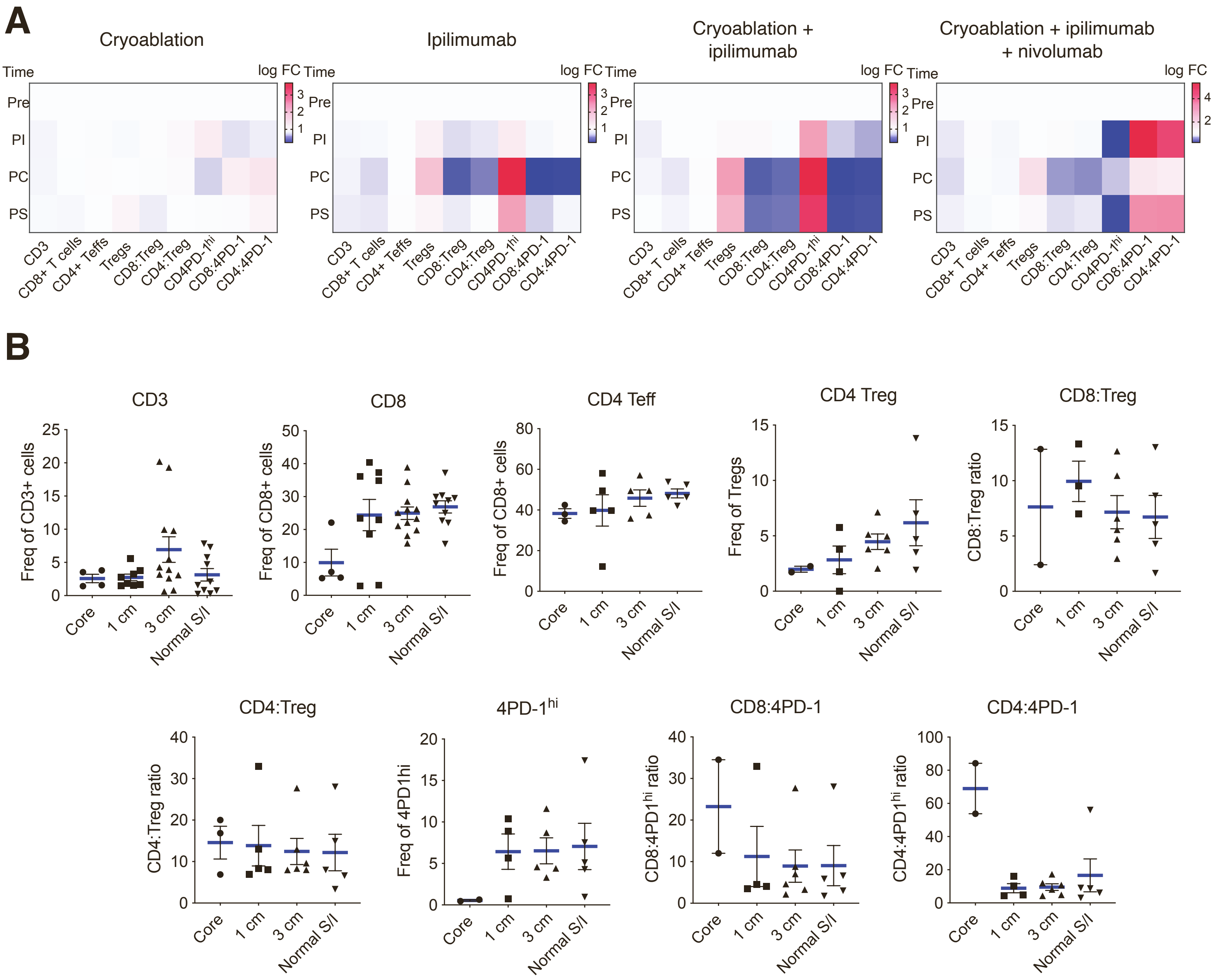
A
Cryoablation
Ipilimumab
Cryoablation + ipilimumab
Cryoablation + ipilimumab + nivolumab
Time
log FC
Pre
PI
PC
PS
CD3
CD8+ T cells
CD4+ Teffs
Tregs
CD8:Treg
CD4:Treg
CD4PD-1hi
CD8:4PD-1
CD4:4PD-1
B
CD3
CD8
CD4 Teff
CD4 Treg
CD8:Treg
Freq of CD3+ cells
Freq of CD8+ cells
Freq of Tregs
CD8:Treg ratio
Core
1 cm
3 cm
Normal S/I
CD4:Treg
4PD-1hi
CD8:4PD-1
CD4:4PD-1
CD4:Treg ratio
Freq of 4PD1hi
CD8:4PD1hi ratio
CD4:4PD1hi ratio

Figure S1. Combination of ipilimumab, nivolumab, and cryoablation reduces CD4+ PD-1^{hi} cells in the periphery, related to Figure 2. A. Heatmaps of T cell populations of the ratio of effector to suppressor T cells in each treatment group. Pre = baseline (pre-treatment), PI = post-immunotherapy, PC = post-cryoablation, PS = post-surgery. Cohort numbers are: cryoablation, n=7; ipilimumab, n=6; cryoablation plus ipilimumab, n= 6; cryoablation plus ipilimumab plus nivolumab, n=5. **B.** Banked single-cell suspensions of tumor-infiltrating lymphocytes isolated from the tumors were analyzed by flow cytometry. Shown are the frequencies of total CD3+, CD8+,–and CD4+ effector (CD4+Foxp3-) T cells, Tregs (CD4+Foxp3+), and 4PD1^{hi} (CD4+Foxp3-PD1^{hi}) T cells at various tumor and tissue sites +/- standard error of samples pooled from 3-5 patients. Normal S/I, normal tissue pooled from superior and inferior samples (A). Statistics were calculated using Student's t test: *p<0.05, **p<0.01, ***p<0.05.

Figure S2

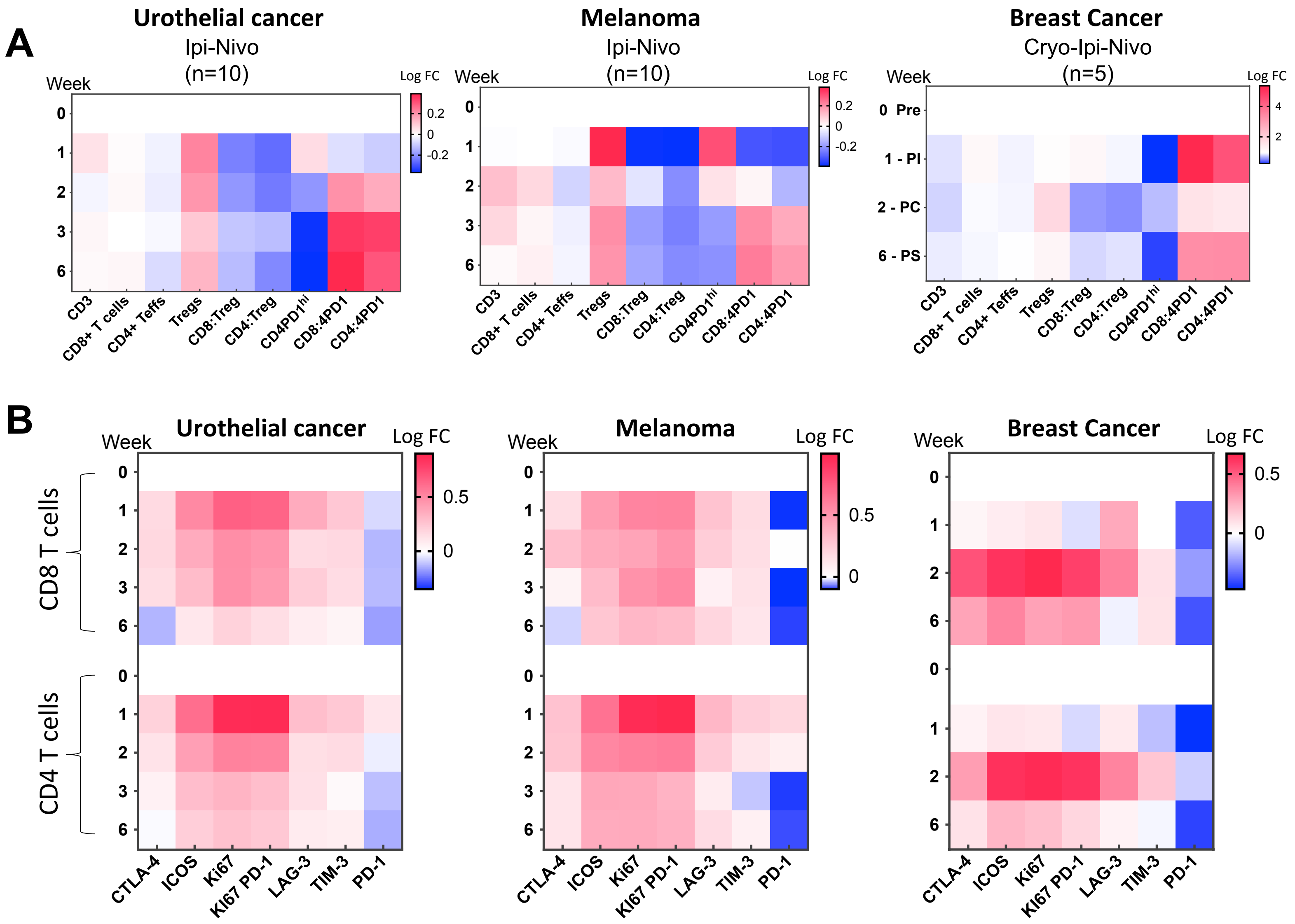

A
Urothelial cancer
Ipi-Nivo
(n=10)
Melanoma
Ipi-Nivo
(n=10)
Breast Cancer
Cryo-Ipi-Nivo
(n=5)
Week
Log FC
0 Pre
1 - PI
2 - PC
6 - PS
CD3
CD8+ T cells
CD4+ Teffs
Tregs
CD8:Treg
CD4:Treg
CD4PD1hi
CD8:4PD1
CD4:4PD1
B
Urothelial cancer
Melanoma
Breast Cancer
CD8 T cells
CD4 T cells
CTLA-4
ICOS
Ki67
KI67 PD-1
LAG-3
TIM-3
PD-1

Figure S2: Ipilimumab plus nivolumab in urothelial cancer (UC) and melanoma as compared with cryoablation plus ipilimumab plus nivolumab in breast cancer, related to Figure 2. Flow cytometry data was obtained from published work[21] on patients with UC and melanoma treated with ipilimumab plus nivolumab and a similar analysis was performed as in the cohort of patients with breast cancer cohort receiving cryoablation plus ipilimumab plus nivolumab. **A.** Heatmaps of T cell populations of the ratio of effector to suppressor T cells in each treatment group. Data is represented as the average $\log_{10}$ fold-change (log FC) relative to baseline (t=0, Pre) for each time point. **B.** Heatmaps of expression of T cell activation markers in CD4+ T effector (Teff) cells and CD8+ T cells in each cohort.

Figure S3

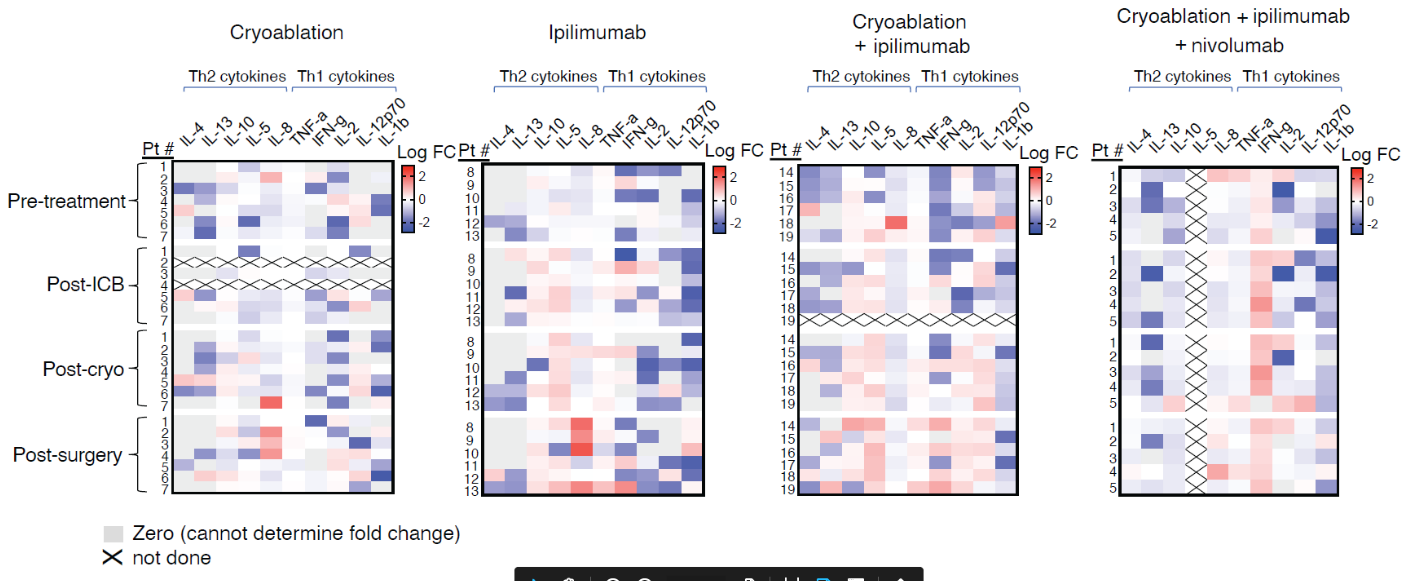

Cryoablation
Ipilimumab
Cryoablation + ipilimumab
Cryoablation + ipilimumab + nivolumab
Th2 cytokines
Th1 cytokines
Pt #
IL-4
IL-13
IL-10
IL-5
IL-8
TNF-a
IFN-g
IL-2
IL-12p70
IL-1b
Log FC
Pre-treatment
Post-ICB
Post-cryo
Post-surgery
Zero (cannot determine fold change)
not done

Figure S3. Dual immune checkpoint blockade with cryoablation induces a distinct cytokine response from that to ipilimumab and/or cryoablation, related to Figure 2. Heatmaps of serum Th1 and Th2 cytokines in serum in each treatment group. Data for each time point are represented as the average of $\log_{10}$ fold change (FC) relative to the baseline levels (pg/ml) for each cytokine. Cohort numbers are: cryoablation, n=7; ipilimumab, n=6; cryoablation plus ipilimumab, n= 6; cryoablation plus ipilimumab plus nivolumab, n= 5.

Figure S4

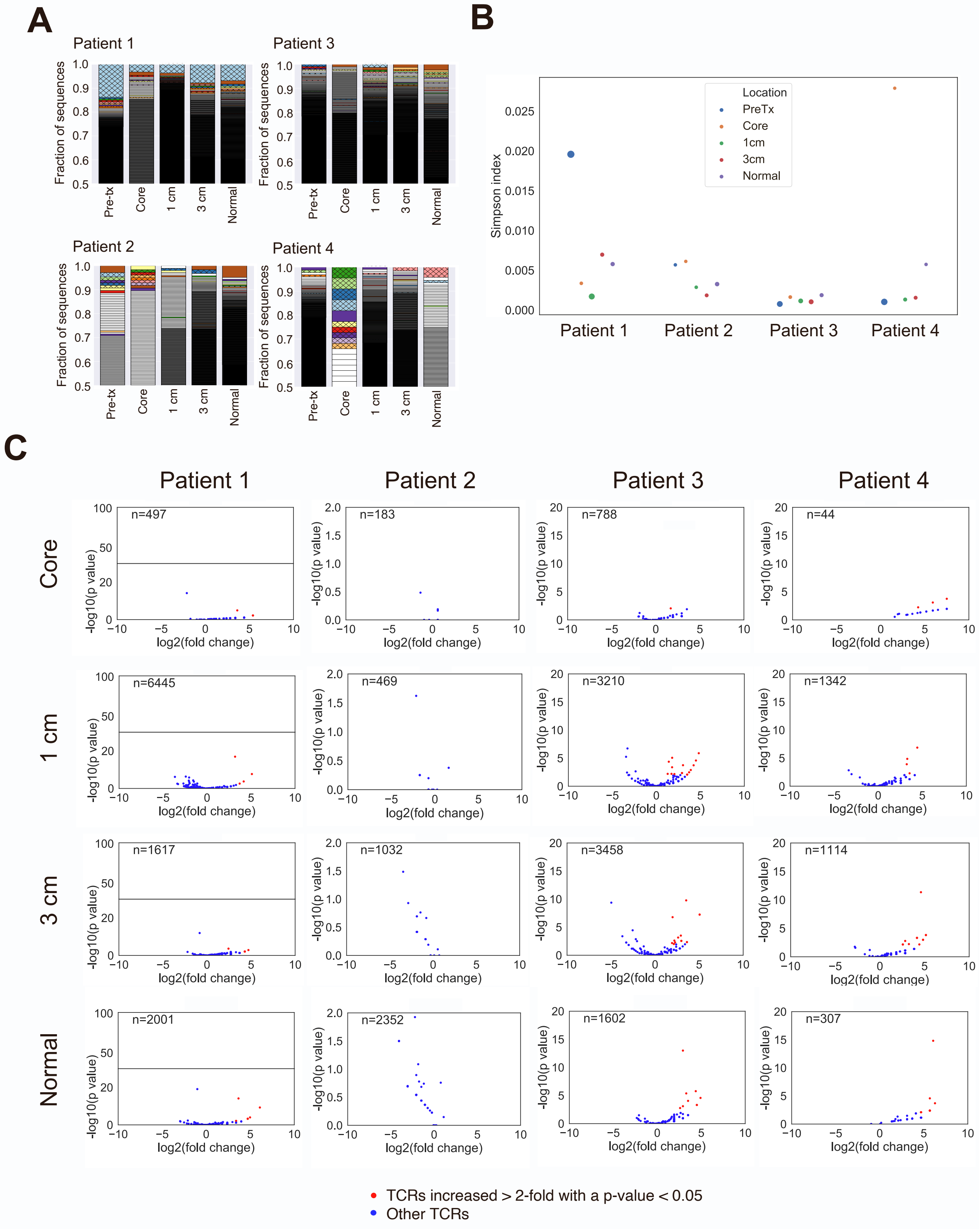

A
Patient 1
Patient 3
Patient 2
Patient 4
Fraction of sequences
Pre-tx
Core
1 cm
3 cm
Normal
B
Simpson index
Location
PreTx
Core
1cm
3cm
Normal
Patient 1
Patient 2
Patient 3
Patient 4
C
Patient 1
Patient 2
Patient 3
Patient 4
Core
1 cm
3 cm
Normal
-log10(p value)
log2(fold change)
n=497
n=183
n=788
n=44
n=6445
n=469
n=3210
n=1342
n=1617
n=1032
n=3458
n=1114
n=2001
n=2352
n=1602
n=307
TCRs increased > 2-fold with a p-value < 0.05
Other TCRs

Figure S4. T cell receptor (TCR) sequencing analysis of tumor samples pre- and post-treatment, related to Figure 3. DNA was isolated from formalin-fixed paraffin-embedded curls of pre- and post-treatment tumor sections. TCRs were sequenced by Adaptive Biotechnologies. **A.** Frequencies of T cell clones in the tumor ranked by abundance. Color coding for each patient matches that for T cell clones in blood (as in Figure 3A). **B.** Simpson index for each time point in the tumor. **C.** Volcano plots of $\log_2$ fold change (Fc) vs. negative $\log_{10}$ p-value of data compared with pre-treatment in blood. Lines through the y-axis indicate a change in p-value scale.

Figure S5

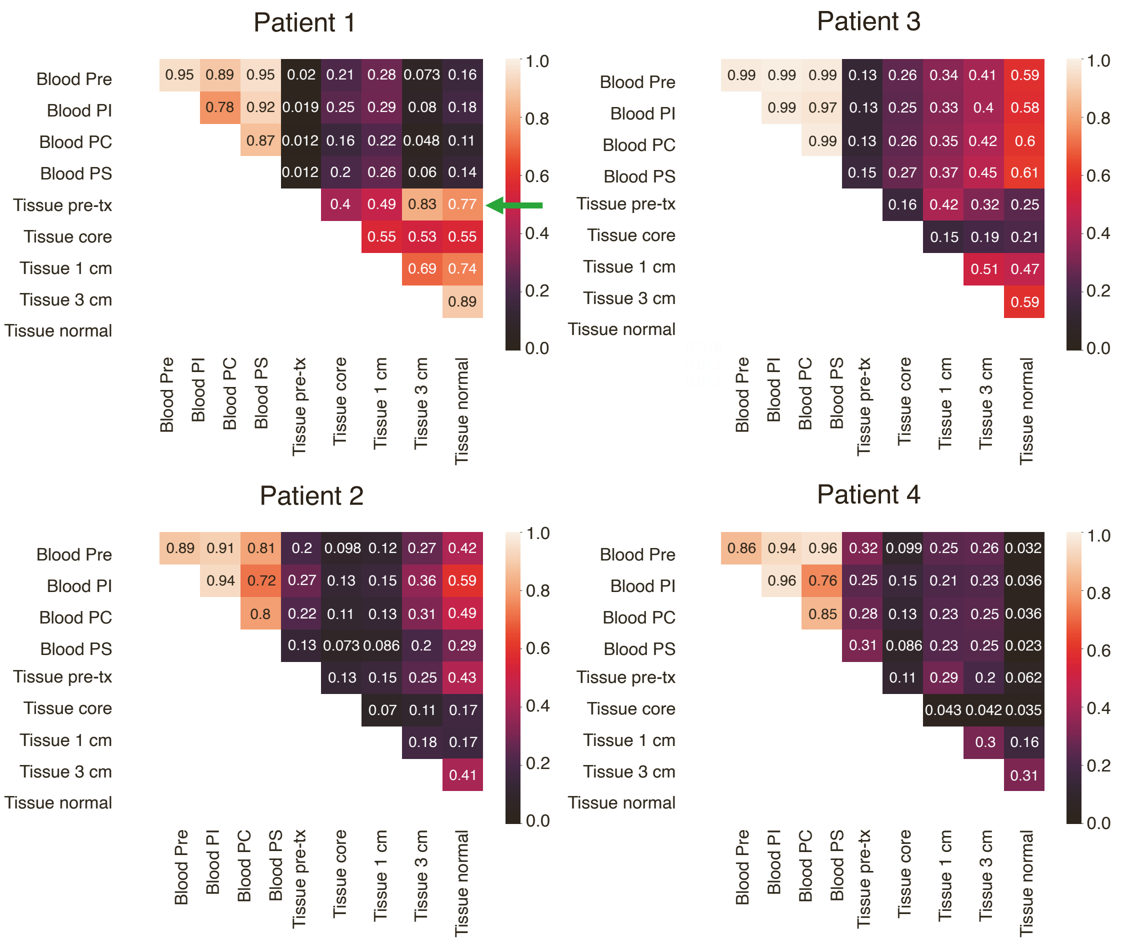
Patient 1
Patient 3
Patient 2
Patient 4
Blood Pre
Blood PI
Blood PC
Blood PS
Tissue pre-tx
Tissue core
Tissue 1 cm
Tissue 3 cm
Tissue normal
0.0
0.2
0.4
0.6
0.8
1.0

Figure S5. Limited overlap between T cell receptor (TCR) clones in blood and tumor, related to Figure 3. Heatmaps of the calculated Morisita overlap index (values in each box) between blood and tissue; 1 reflects identical TCR clones and 0 represents no overlap.

Table S1. Breakdown of the timeline for sample collection of each patient, related to Figure 1

**Abbreviation:**		**Pre**	**PI**	**PC**	**PS**
		**Pre-treatment**	**Post-immunotherapy**	**Post-cryoablation**	**Post-surgery**
**Pt #**	**1**	0	1.1	2.3	7.0
	**2**	0	0.9	1.9	6.9
	**3**	0	1.1	2.6	6.7
	**4**	0	1.0	2.0	6.4
	**5**	0	1.0	1.9	7.1
**Average**		**0.0**	**1.0**	**2.1**	**6.8**

Timeline is shown in weeks.

Table S2. Number of productive T cell receptors (TCRs) per patient sample, related to Figure 3

Patient	Sample ID	Assay	Sample Type	Timepoint	Location*	Treatment	Productive TCRs**
1	16-495-1A	TCRB	Blood	0	N/A	No Tx	131039
	16-495-1B	TCRB	Blood	1	N/A	Ipi-Nivo	226404
	16-495-1C	TCRB	Blood	2	N/A	Cryo-Ipi-Nivo	59006
	16-495-1D	TCRB	Blood	6	N/A	Cryo-Ipi-Nivo	47039
	16-495-1TA	TCRB	FFPE Tissue	1	PreTx	Ipi-Nivo	10503
	16-495-1TB	TCRB	FFPE Tissue	2	Core	Cryo-Ipi-Nivo	497
	16-495-1TC	TCRB	FFPE Tissue	2	1 cm – 1	Cryo-Ipi-Nivo	5511
	16-495-1TD	TCRB	FFPE Tissue	2	1 cm – 2	Cryo-Ipi-Nivo	934
	16-495-1TE	TCRB	FFPE Tissue	2	3 cm – 1	Cryo-Ipi-Nivo	521
	16-495-1TF	TCRB	FFPE Tissue	2	3 cm – 2	Cryo-Ipi-Nivo	1096
	16-495-1TG	TCRB	FFPE Tissue	2	Normal sup	Cryo-Ipi-Nivo	835
	16-495-1TH	TCRB	FFPE Tissue	2	Normal inf	Cryo-Ipi-Nivo	571
	16-495-1TI	TCRB	FFPE Tissue	2	Normal fib	Cryo-Ipi-Nivo	595
2	16-495-2A	TCRB	Blood	0	N/A	No Tx	38693
	16-495-2B	TCRB	Blood	1	N/A	Ipi-Nivo	165215
	16-495-2C	TCRB	Blood	2	N/A	Cryo-Ipi-Nivo	72961
	16-495-2D	TCRB	Blood	6	N/A	Cryo-Ipi-Nivo	47647
	16-495-2TA	TCRB	FFPE Tissue	1	PreTx	Ipi-Nivo	281
	16-495-2TB	TCRB	FFPE Tissue	2	Core	Cryo-Ipi-Nivo	183
	16-495-2TC	TCRB	FFPE Tissue	2	1 cm – 1	Cryo-Ipi-Nivo	323
	16-495-2TD	TCRB	FFPE Tissue	2	1 cm – 2	Cryo-Ipi-Nivo	146
	16-495-2TE	TCRB	FFPE Tissue	2	3 cm – 1	Cryo-Ipi-Nivo	702
	16-495-2TF	TCRB	FFPE Tissue	2	3 cm – 2	Cryo-Ipi-Nivo	330
	16-495-2TG	TCRB	FFPE Tissue	2	Normal sup	Cryo-Ipi-Nivo	237
	16-495-2TH	TCRB	FFPE Tissue	2	Normal inf	Cryo-Ipi-Nivo	2115
3	16-495-5A	TCRB	Blood	0	N/A	No Tx	146060
	16-495-5B	TCRB	Blood	1	N/A	Ipi-Nivo	248639
	16-495-5C	TCRB	Blood	2	N/A	Cryo-Ipi-Nivo	131950
	16-495-5D	TCRB	Blood	6	N/A	Cryo-Ipi-Nivo	152416
	16-495-5TA	TCRB	FFPE Tissue	1	PreTx	Ipi-Nivo	6050
	16-495-5TB	TCRB	FFPE Tissue	2	Core	Cryo-Ipi-Nivo	788
	16-495-5TC	TCRB	FFPE Tissue	2	1 cm – 1	Cryo-Ipi-Nivo	1050
	16-495-5TD	TCRB	FFPE Tissue	2	1 cm – 2	Cryo-Ipi-Nivo	2160
	16-495-5TE	TCRB	FFPE Tissue	2	3 cm – 1	Cryo-Ipi-Nivo	2358
	16-495-5TF	TCRB	FFPE Tissue	2	3 cm – 2	Cryo-Ipi-Nivo	1100
	16-495-5TG	TCRB	FFPE Tissue	2	Normal	Cryo-Ipi-Nivo	1602
	16-495-5TH	TCRB	FFPE Tissue	2	Contralateral	Cryo-Ipi-Nivo	8376
4	16-495-6A	TCRB	Blood	0	N/A	No Tx	82943
	16-495-6B	TCRB	Blood	1	N/A	Ipi-Nivo	250704
	16-495-6C	TCRB	Blood	2	N/A	Cryo-Ipi-Nivo	86321
	16-495-6D	TCRB	Blood	6	N/A	Cryo-Ipi-Nivo	141680
	16-495-6TA	TCRB	FFPE Tissue	1	PreTx	Ipi-Nivo	7925
	16-495-6TB	TCRB	FFPE Tissue	2	Core	Cryo-Ipi-Nivo	44
	16-495-6TC	TCRB	FFPE Tissue	2	1 cm - 1	Cryo-Ipi-Nivo	559
	16-495-6TD	TCRB	FFPE Tissue	2	1 cm - 2	Cryo-Ipi-Nivo	783
	16-495-6TE	TCRB	FFPE Tissue	2	3 cm - 1	Cryo-Ipi-Nivo	366
	16-495-6TF	TCRB	FFPE Tissue	2	3 cm - 2	Cryo-Ipi-Nivo	748
	16-495-6TG	TCRB	FFPE Tissue	2	Normal	Cryo-Ipi-Nivo	307

TCRB, Adaptive Biotechnologies hsTCRBv4 assay; FFPE, formalin-fixed paraffin-embedded; sup, superior; inf, inferior; fib, fibrous; Tx, treatment; cryo, cryoablation; ipi, ipilimumab; nivo, nivolumab

**Tissue location is tumor unless otherwise noted; cm denotes centimeters from tumor core*

***TCR clone abundance tables are provided as tsv files in supplemental information*
